# Cellular EMT-status governs contact guidance in an electrospun TACS-mimicking *in vitro* model

**DOI:** 10.1016/j.mtbio.2024.101401

**Published:** 2024-12-10

**Authors:** Lorenz Isert, Mehak Passi, Benedikt Freystetter, Maximilian Grab, Andreas Roidl, Christoph Müller, Aditi Mehta, Harini G. Sundararaghavan, Stefan Zahler, Olivia M. Merkel

**Affiliations:** a Pharmaceutical Technology and Biopharmaceutics, Department of Pharmacy, Ludwig-Maximilians-University München, Munich, Germany; b Pharmaceutical Biology, Department of Pharmacy, Ludwig-Maximilians-Universität München, Munich, Germany; c Department of Cardiac Surgery, Ludwig Maximilians University München, Munich, Germany; d Pharmaceutical Biotechnology, Department of Pharmacy, Ludwig-Maximilians-Universität München, Munich, Germany; e Center of Drug Research, Department of Pharmacy, Ludwig-Maximilians-Universität München, Munich, Germany; f Department of Biomedical Engineering, Wayne State University, Detroit, MI, USA

## Abstract

In this study, an advanced nanofiber breast cancer *in vitro* model was developed and systematically characterized including physico-chemical, cell-biological and biophysical parameters. Using electrospinning, the architecture of tumor-associated collagen signatures (TACS5 and TACS6) was mimicked. By employing a rotating cylinder or static plate collector set-up, aligned fibers (TACS5-like structures) and randomly orientated fibers (TACS6-like structures) fibers were produced, respectively. The biocompatibility of these fibers was enhanced by collagen coating, ensuring minimal toxicity and improved cell attachment. Various breast cancer cell lines (MCF7, HCC1954, MDA-MB-468, and MDA-MB-231) were cultured on these fibers to assess epithelial-to-mesenchymal transition (EMT) markers, cellular morphology, and migration.

Aligned fibers (TACS5) significantly influenced EMT-related changes, promoting cellular alignment, spindle-shaped morphology and a highly migratory phenotype in mesenchymal and hybrid EMT cells (MDA-MB-468, MDA-MB-231). Conversely, epithelial cells (MCF7, HCC1954) showed limited response, but - under growth factor treatment - started to infiltrate the fibrous scaffold and underwent EMT-like changes, particularly on TACS5-mimicks, emphasizing the interplay of topographical cues and EMT induction.

The biophysical analysis revealed a clear correlation between cellular EMT status and cell mechanics, with increased EMT correlating to decreased total cellular stiffness. Cancer cell mechanics, however, were found to be dynamic during biochemical and topographical EMT-induction, exceeding initial stiffness by up to 2-fold. These findings highlight the potential of TACS5-like nanofiber scaffolds in modeling the tumor microenvironment and studying cancer cell behavior and mechanics.

## Graphical abstract

**Figure:**
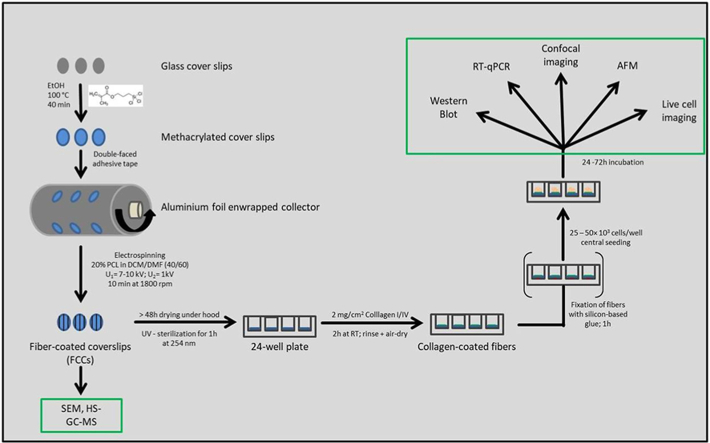

## 1 Introduction

According to the International Agency for Research on Cancer, female breast cancer (BC) had 2.3 million new cases in 2020, making it the most prevalent cancer worldwide [1]. About 7 % of patients die, mainly due to metastasis. Optimizing diagnostics and discovering new prognostic factors are crucial, alongside well-accepted clinico-pathological factors like biomarker status, patient age, comorbidity, tumor size, grade, and lymph node involvement [2].

Recently, the histological features of the acellular tumor microenvironment (TME) have gained attention. High breast tissue density, due to increased collagen, is a significant risk factor for BC. Tumor-associated collagen signatures (TACS) may serve as prognostic markers [3], correlating with tumor grade and prognosis [[4], [5], [6]]. Notably, TACS5 (aligned collagen fibers) and TACS6 (randomly aligned fibers) at the tumor's invasive front strongly correlate with poor prognosis [5].

These collagen signatures might act as “migration highways” [7], facilitating cancer cell dissemination. Cancer cells respond to biomechanical cues from the TME, which influence migration and gene expression [[8], [9], [10]]. For instance, cells prefer migrating parallel to topographical features [11], impacting cell morphology and migration efficiency [12]. Matrix alignment, more than stiffness, enhances migration for BC cells by promoting directional persistence [13,14].

Topography-based contact guidance depends on cell-cell interactions. Epithelial clusters respond less to topographical alignment than single cells [15]. This underscores the role of EMT, where cancer cells gain mesenchymal traits, enhancing interaction with TME cues. EMT is linked to increased BC malignancy [[16], [17], [18]], suggesting a synergistic relationship with TACS in metastasis.

Bioengineering applications aim to understand the relationship between tissue-relevant topographies and EMT, which could advance BC diagnostics and treatment targets. Electrospinning, a technique producing fiber mats mimicking native ECM, is valuable in biomedicine [19]. Electrospun scaffolds serve as drug delivery systems, cancer biosensors, and 3D *in vitro* models [20,21].

This study established an electrospun 3D *in vitro* BC model using poly (ε-caprolactone) (PCL) fibers to mimic TACS. By culturing BC cell lines on this model, the study addressed how TACS-mediated contact guidance and EMT phenotype interrelate, how EMT/MET-like changes influence contact guidance, and how phenotypic changes affect cellular mechanics. This interdisciplinary approach aimed to elucidate the reciprocal dependence of cancer cells and the TME in tumor progression.

## 2 Material & methods

### 2.1 Materials and cell culture

Polycaprolacton (M_N_ 80.000), 3-(Trimethoxysilyl)propyl methacrylate 98 %, Formaldehyde solution (≥36 %), Dichloromethane (DCM) anhydrous, *N,N*-Dimethylformamide (DMF) anhydrous, 4′,6–diamidino–2-phenylindole dihydrochloride (DAPI), FluorSave reagent, DNase I (recombinant, RNase-free), cOmplete™, EDTA-free Protease Inhibitor Cocktail, Phosphatase Inhibitor Cocktail 2, RIPA buffer, Tris buffered saline powder, Ponceau S Stain, Tween 20, Amersham™ Protran® Western-Blotting-Membrane (nitrocellulose), Eagle's Minimum Essential Medium (EMEM), RPMI-1640 Medium, Dulbecco's modified eagle's medium (DMEM), fetal bovine serum (FBS), Penicillin-Streptomycin (Pen/Strep) solution, Dulbecco's Phosphate Buffered Saline (PBS), trypsin-EDTA solution 0.05 % and 0.25 %, 200 mM of L-glutamine solution, doxycycline hydrochloride (DOX) and dimethyl sulfoxide (DMSO) were purchased from Sigma-Aldrich (Taufkirchen, Germany). GAPDH Monoclonal Antibody (ZG003), LIVE/DEAD™ Cell Vitality Assay Kit (C12 Resazurin/SYTOX™), Green Pierce™ BCA Protein Assay Kit, Novex™ 10 % Tris-Glycine Mini Gels (WedgeWell™ format, 15-well), Novex™ Value™ 4–20 % Tris-Glycine Mini Gels (1.0 mm, 10-well), Page Ruler™ Plus Prestained Protein Ladder 10–250 kDa, Tris Glycin transfer buffer, SuperSignal™ West Pico PLUS Chemiluminescent Substrate, Hoechst 33342, Rhodamine Phalloidin, High capacity cDNA synthesis kit, Power SYBR™ Green PCR Master Mix, PureLink™ RNA Mini Kit and Leibovitz's L-15 Medium and MEM Non-Essential Amino Acids Solution (100X) were purchased from Thermo Fisher Scientific (Darmstadt, Germany). Collagen I (sc-136154), Collagen IV (sc-29010), m-IgGκ BP-HRP (sc-516102), COL1A2 antibody (H-9), Anti-Lamin A/C Antibody (636), Anti-Lamin B1 Antibody (B-10), E-cadherin Antibody (G-10) and Vimentin Antibody (V9) were ordered from Santa Cruz Biotechnology (Dallas, TX, USA). Hs_CDH1_Primer Assay (QT00080143), Hs_SNAI1_Primer Assay (QT00010010), Hs_VIM_Primer Assay (QT00095795), Hs_GAPDH_1_SG QuantiTect Primer Assay (QT00079247) and Hs_XBP1_1_SG QuantiTect Primer Assay (QT00068383) were purchased from Qiagen (Hilden, Germany). Rotiphorese 10× SDS Page, Rotilabo®-Blotting Papers and Methanol (blotting grade) were purchased from Carl Roth (Karlsruhe, Germany). rh-TGF-β 1 (Transforming Growth Factor beta 1) and rh-EGF (Epidermal Growth Factor) were acquired from ImmunoTools (Friesoythe, Germany). Laemmli loading buffer (4×) and round glass coverslips (Ø 13 mm) were purchased from VWR (Ismaning, Germany). HyClone trypan blue solution 0.4 % in phosphate-buffered saline was obtained from FisherScientific (Darmstadt, Germany). Injection Luer Lock needle, blunt end (Ø 0.7 × 30 mm) was purchased from Unimed S.A (Lausanne, Switzerland). Human CD44s Pan Specific Antibody was purchased from R&D Systems (Minneapolis, MN, USA). Braun Omnifix® Syringes (20 mL, Luer) were purchased from Neolab (Heidelberg, Germany).

MCF7 (and MCF7 miR200c_KO) cells, a Luminal A breast cancer cell line, were cultured in EMEM supplemented with 10 % FBS, 1× Pen/Strep, 1× MEM Non-Essential Amino Acids Solution and 2 mM L-glutamine. The HER2-positive breast cancer cell line HCC1954 was grown in RPMI-1640 Medium supplemented with 10 % FBS and 1× Pen/Strep. MDA-MB-231 (and MDA-MB-231 i-miR200c) cells, a triple negative breast cancer (TNBC) cell line, were cultured in high glucose (4500 mg/L) DMEM, and 10 % FBS, 1× Pen/Strep and 2 mM glutamine were added to the medium. For miRNA induction in MDA-MB-231 i-miR200c cells, the medium was equipped with 5 μg/mL doxycycline hydrochloride which was replenished every 48 h. The latter three cell lines were cultured in a humidified atmosphere with 5 % CO_2_ at 37 °C. The second TNBC cell line MDA-MB-468 (DSMZ, Braunschweig, Germany) was grown in L-15 Medium supplemented with 20 % FBS and 1× Pen/Strep. These cells were grown in a humidified incubator with 0 % CO_2_ at 37 °C.

### 2.2 Electrospinning of TACS-like structures: set-up and processing of cell culture inserts

The used electrospinning system was custom made by the Laboratory for Tissue Engineering and Cardiovascular Medical Technology of the Clinic of Cardiac Surgery at the Ludwig-Maximilian-University in Munich. Briefly, the test chamber included air conditioning for organic solvents to evaporate and contained molecular sieves to reduce humidity within the chamber. Either a static aluminum plate or a dynamic rotating cylinder was used as collectors to produce unaligned or aligned fibers, respectively. The cylinder (Ø 150 mm) itself was assembled out of 3D-printed constructs using the Keyence Agilista 3200W printer. The use of non-conductive AR – M2 (Keyence Corp, Osaka JP) acrylic polymer necessitated an additional wrapping step with aluminum foil to enable electrostatic attraction. A NEMA 17 motor (Nanotec Electronic GmbH, Feldkirchen, Germany) connected to a computer via an associated control card was used to drive the cylinder. The speed and direction of rotation were set using the “Plug & Drive Studio” (Nanotec) software. A high-voltage power supply from iseg GmbH (Radeberg, Germany) with a voltage range of 0–30 kV (positive and negative) was used to generate the electric field. The voltage supply was coupled to the needle and the collector by means of a high-voltage cable. The flow rate of the polymeric solution was controlled by a laboratory syringe pump (Fusion 100, Chemyx Inc., Stafford, TX, USA).

**Fig. 1:**
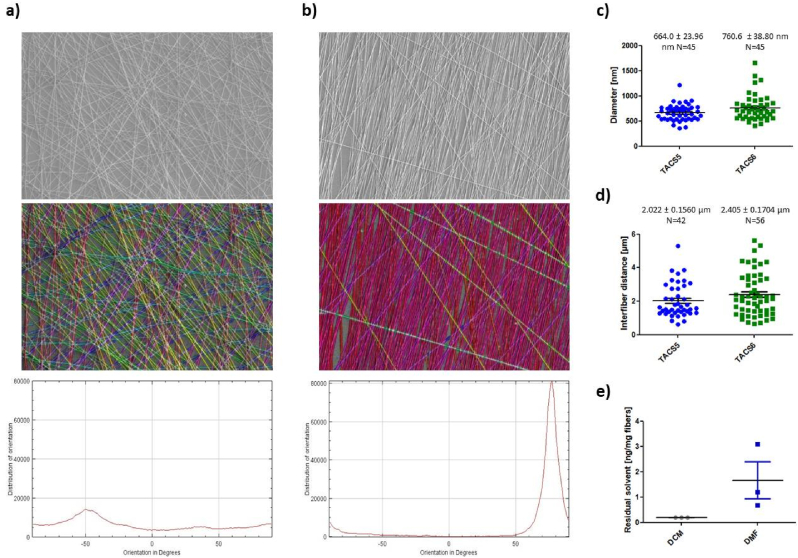
(a, b) SEM image analysis: Upper panel shows representative SEM images of (a) TACS6- and (b) TACS5-like structures (10.000× magnification); mid panel highlights single fiber orientations with same colors representing same fiber orientation; lower panel depicts distribution intensity of fiber orientation (−90°–90°). (c) Fiber diameters and (d) inter-fiber distances of TACS5- and TACS6-like structures analyzed with Fiji software. (e) Residual amount of organic solvents (DCM and DMF) in ng/mg fiber were analyzed with HS-GC-MS. Errors indicate standard error of mean (SEM).

PCL (20 g) was dissolved in 100 mL of DCM/DMF (40/60) 24 h prior to the electrospinning process. The solution was stirred over night at 400 rpm covered with aluminum foil to avoid evaporation of the organic solvents and degradation of PCL. To increase adhesion of fibers to glass coverslips, coverslips were incubated for 5 min in 4 % (*v/v*) of 3-(Trimethoxysilyl)propyl methacrylate in absolute ethanol. The solution was removed and rinsed 3 times with pure ethanol. Coverslips were subsequently baked at 100 °C for 40 min. To produce cell culture inserts, optimized (methacrylated) coverslips were mounted either on the plate collector or rotating cylinder using double-sided adhesive tape as can be seen in Fig. S1a. The rotation speed was set to 1800 rpm. A 20 mL syringe was loaded with the polymer (PCL) solution and connected to the syringe pump. The flow rate was set to 1.0 mL/h. The high-voltage supply (+7–10 kV) was connected to a needle (Ø 0.7 × 30 mm) placed 21–23 cm away from the collector. Opposite, constant charge was applied to the collector (-1 kV). In case of the rotating cylinder, a sliding contact was used. To assure constant fiber deposition during the 10 min of the electrospinning process, Taylor cone formation was monitored at the tip of the nozzle. As shown in Fig. 1, fiber-coated coverslips (FCCs) were consequently either subjected to SEM analysis or further processed to fulfill cell culture requirements. To remove excess amounts of organic solvents, FCCs were placed in a chemical hood for 48 h. Afterwards FCCs were sterilized using UV-light for 1 h and thereafter placed into a 24-well plate under sterile conditions where they were exposed to UV-light for another hour. Subsequently, for cell culture work, FCCs were coated with collagen (I/IV) as described elsewhere [22]. Methacrylated glass coverslips coated with collagen were used as reference samples (referred to as blank or conventional) for all methods, if not stated otherwise. For AFM and live-cell imaging purposes, collagen-coated FCCs were fixed onto the surfaces of culture dishes. Therefore, 3 μL of silicon-based glue were placed in the center of the bottom side of the FCCs and let dry under sterile conditions for 1 h prior to cell seeding. For the seeding process, 25.000–50.000 cells were suspended in 200 μL of the respective medium and then slowly pipetted on the scaffold starting at the center and spirally approaching the margin of the FCC with a blob emerging and covering the entire FCC. After 15 min of incubation, allowing cells to sediment onto the scaffold, each well was filled up with pre-warmed medium to 1 mL and subjected to various analyses.

### 2.3 Scanning electron microscopy (SEM)

The topography of TACS5- and TACS6-like scaffolds (FCCs) were examined by scanning electron microscopy (SEM) using a JSM-6510LVLGS, 25 kV (JEOL, Peabody, MA, USA). For imaging, scaffolds were sprinkled on a stub covered with double-sided carbon tape and sputter-coated with gold (Ernest Fullan) under vacuum for 60 s. Topographic features including fiber orientation, diameter and density were analyzed from the SEM images by processing with the Fiji imaging software (Version 2.30/1.53q) [23]. Fiber orientation and distribution of orientation were analyzed from representative samples of each collector type using the OrientationJ plug-in (written by Daniel Sage). Fibers represented with the same color indicate same directionality. Fiber diameters and inter-fiber distances were manually calculated from 10.000× images taken from samples of 2 independent electrospinning runs per collector type.

### 2.4 GC analysis – residual solvents

Residual DCM and DMF content was analyzed by static headspace-gas chromatography-mass spectrometry (HS-GC-MS). An Agilent Technologies 7890B gas chromatograph (Waldbronn, Germany), equipped with an Agilent J&W DB-624 UI ultra-inert capillary column (6 % cyanopropyl phenyl and 94 % polydimethylsiloxane) 30 m × 0.25 mm × 1.4 μm and an Agilent Technologies 7010B triple quadrupole detector with high efficiency source (HES) was used for analysis. Helium (99.999 %) was used as mobile phase. DCM (HPLC grade), 1,2-dichloroethane (DCE, purity 97 %), DMF (purity HPLC grade), DMF-d7 (purity 99.5 %), and DMSO (purity HPLC grade) were purchased from Sigma-Aldrich (Schnelldorf, Germany). As samples, 15 mg fiber mesh (obtained from three independent electrospinning runs, respectively) were filled into a 20 mL headspace vial, 10 μL of DMF-d7 and DCE (each 10 μg/mL in DMSO) as internal standards were added, and the vial was closed tightly. After sealing, the sample was analyzed by HS-GC-MS (see Table S1). The MS was operated in single ion monitoring mode (SIM; EI 70 eV). The retention times and characteristic ions of DCM (5.6 min, *m*/*z* 84.1), DCE (internal standard; 8.2 min, *m*/*z* 62.1), DMF (9.8 min, *m*/*z* 73.2), and DMF-d7 (internal standard; 9.9 min, *m*/*z* 80.2) were used for identification and quantification. For detailed information on HS-GC-MS conditions, see Table S1 in the supplements.

### 2.5 Cell viability

The cell viability of cells growing on TACS5- and TACS6-like (data not shown) structures was assessed by applying the LIVE/DEAD™ Cell Vitality Assay Kit (Invitrogen™) and subsequent fluorescence microscopy. For each sample, 35.000 MDA-MB-231 cells were seeded on collagen I – coated tissue plates, uncoated FCCs and coated FCCs. All samples were cultivated for 72 h and then stained according the manufacturer's protocol. Untreated cells on conventional culture dishes served as positive (live/red) control, whereas negative (dead/green) control cells were incubated for 30 min with ethanol 70 % (*v/v*) prior to the analysis. Fluorescence images were recorded with the Keyence BZ81000 Fluorescence microscope (Keyence, Osaka, Japan). Images of transmitted light, green and red channel were taken simultaneously with a 20× objective and further analyzed with Fiji software. Percentages of living/dead cells was calculated in duplicates (with each sample >100 cells), counting red/green fluorescence signals (cells) of the respective channels.

### 2.6 Confocal scanning microscopy – morphological changes

Confocal laser scanning microscopy was used to assess morphological changes across different cell lines growing on glass coverslips, TACS5- and TACS6-like structures. As performed with the FCCs, coverslips were coated with collagen I and IV. Thereinafter, 35.000–50.000 cells of the respective cell lines were seeded in 24-well plates following the protocol described above. In case of growth factor (GF) treatment, cells were allowed to attach within the first 4 h. Medium was then removed and replenished with GF-containing medium. Samples were either supplied with 10 ng/mL of TGF-β1 (MCF7 and HCC1954) or 25 ng/mL of EGF (MDA-MB-468). DMEM medium used for miR200c induction in modified MDA-MB-231 cells contained 5 μg/mL of doxycycline hydrochloride (DOX). After 72 h of incubation, wells were washed 3 times with PBS before cells were fixed for 15 min with a 4 % formaldehyde solution. To stain the actin cytoskeleton, cells were incubated with 8.25 μM rhodamine-phalloidin solution for 40 min. Hereinafter, cells were washed another 3 times with PBS. Nuclei were stained with a 0.5 μg/mL DAPI solution for 10 min. Finally, after an additional washing step, samples were mounted on glass slides using FluorSave and stored at 4 °C until the next day. Fluorescence images were acquired using a confocal laser scanning microscope (Leica SP8 inverted, Software: LAS X, Leica microsystems GmbH, Wetzlar, Germany) equipped with a HC PL APO CS2 63×/1.40 oil immersion objective. Diode lasers (405 nm) and a semiconductor laser OPSL (552 nm) were chosen for excitation. Emission was detected in blue (PMT1: 410–520 nm) and yellow (PMT2: 560 nm–760 nm), respectively. Images were further processed with Fiji image analysis software. Nuclear circularity was calculated as $C_{N}=\frac{4\pi A}{P^{2}}$, (n > 30; A: area, P: perimeter), and the cellular aspect ratio as $A_{R}=\frac{d_{\min}}{d_{\max}}$, (n > 15). The axes (d_min_ and d_max_) were drawn manually. Results are given as Whiskers Plots with error bars indicating minimal and maximal values. One-way ANOVA with Bonferroni multiple comparison test was performed in GraphPad Prism software version 5.00 (Graph Pad Software, La Jolla, CA, USA) to calculate P-values at a 95 % confidence interval.

### 2.7 Live cell imaging – motility and contact guidance

Live cell imaging was performed using the Eclipse Ti inverted microscope (Nikon, Düsseldorf, Germany) with a 4/10 phase contrast objective and a charge-coupled device camera (DS-Qi1Mc; Nikon). After 48–72 h of incubation, nuclei of cancer cells were stained with Hoechst 33342 dye (Invitrogen™) according to the manufacturer's protocol. Briefly, cells were washed once with PBS and subsequently incubated for 20 min in the cell culture incubator in 300 μL of 0.5 μg/mL dye in PBS. Afterwards cells were washed another 2 times, and 1 mL of the respective medium was replenished. The 24-well plates were inserted into a 37 °C heating and incubation system that was flushed with actively mixed 5 % CO2 (0 % in case of MDA-MB-468 cells) at a rate of 10 L/h, and the humidity was kept at 80 % to prevent dehydration of cells. The cells were imaged in bright-field, and the nuclei were detected at 405 nm using the integrated fluorescence LED. Time-lapse videos were taken with a time interval of 5 min between images over 24 h.

### 2.8 Tracking of single-cell trajectories

For single-cell trajectories, the position of nuclei was analyzed using the TrackMate plugin within the Fiji software [24]. To extract nuclear circularity trajectory, the image series of the 405 nm channel was manually processed (*Enhance contrast (5.0* *%)* ≫ 2× *Smooth* ≫ *Convert to mask*) and further analyzed with “Mask detector” function of the TrackMate plugin (see Table 1).

**Table 1:** Description of variables calculated with the “Track analyzer” function of the TrackMate plugin (Fiji software) [24], which was used to evaluate differences in contact guidance amongst cell lines and treatments.

Track displacement	Confinement Ratio	Mean directional change
Measure of the distance [μm] between first (0 h) and last spot (24 h) of a track. Indicative for how far a cell has migrated away from its starting point but not total distance it has traveled.	= Linearity of forward progression = $\frac{track\;displacement\;\left[\mu m\right]}{total\;distance\;\left[\mu m\right]}$ The confinement ratio indicates how efficient a cell displaced from its origin.	Measures the angle between two succeeding links, which is then averaged over all links of a track. Low values indicate high consistency of directed migration/migratory persistence and *vice versa*.

### 2.9 EMT-marker expression – RT-qPCR

In 24-well plates, 40.000 cells of the respective cell line were seeded in triplicates. Importantly, to allow for the best possible comparison, all cells were seeded on collagen I coated coverslips or coated FCCs. GF and DOX supply was performed as described above. After 72 h incubation, cells were washed 3-times with PBS. Subsequently, 300 μL of ethanol-containing lysis buffer was added for 15 min. In order to maximize the amount of RNA for analysis, triplicates were pooled together prior to the RNA isolation step. Total RNA was isolated using the PureLink RNA mini kit according to the manufacturer's protocol with additional DNAse I digestion. Subsequently, 500 ng of RNA was used to synthesize cDNA using the High capacity cDNA synthesis kit. In the following, E-cadherin (CDH1)-, Snail (SNAI1)-, Vimentin (VIM)-, and XBP1-specific primers were used to amplify and quantify RNA using Power SYBR™ Green PCR Master Mix and the qTOWER real-time PCR thermal cycler (Analytik Jena, Jena, Germany). *C*_*t*_ values were normalized to GAPDH RNA expression, and delta C_t_ values were calculated for the comparison. One-way ANOVA with Bonferroni multiple comparison test was performed in GraphPad Prism software (Graph Pad Software, La Jolla, CA, USA) to calculate P-values at a 95 % confidence interval (n = 4).

### 2.10 EMT-marker protein levels – Western blotting

In 24-well plates, 40.000 cells of the respective cell line were seeded in triplicates. Importantly, to have the best comparison possible, cells were seeded on collagen I coated coverslips or coated FCCs (here: TACS5). DOX was supplied as described above. After 72 h incubation, cells were washed 3 times with PBS prior to cell lyses. Subsequently, 60 μL of proteinase- and phosphatase-inhibitor containing RIPA buffer was added to one well (of a triplicate) and cells were kept on ice for 20 min. Thereinafter, wells were thoroughly scraped and the extract was transferred to the next well of a triplicate. This step was repeated again for the last well of a triplicate (including 20 min of incubation). This step was necessary to maximize the protein yield. Finally, pooled triplicates were transferred into 1.5 mL microcentrifuge tubes. After a 10 min centrifugation step at 4 °C, total protein concentration was assessed according to the manufacturer's protocol (Pierce™ BCA Protein Assay Kit). Gels were loaded with 30 μg protein per sample, and electrophoresis was run for 90 min at 120 mV. Subsequent to 1 h of protein transfer at 100 mV, blots were washed, blocked and incubated overnight using CD44s-, CDH1-, VIM- and GAPDH-specific antibodies. HRP-bound secondary antibody was then added for 1 h under exclusion of light before blots were developed.

### 2.11 Atomic force microscopy (AFM)

All stiffness measurements were performed on a JPK Nano Wizard 4 in cell culture media using the AFM's contact mode (force spectroscopy mode). Tipless MLCT-D cantilevers (silicon nitride, resonance frequency 15 kHz, spring constant 0.03 N/m) glued to a glass bead (diameter microscopically determined) were used to indent the cells. These were calibrated with the contact-free method in liquid. The following parameters were used: set point 1 nN, z-length 10 μm and velocity 5 μm/s. The Petri dish was mounted onto the AFM stage and the cells were kept in cell culture media. A digital petri dish heater attached to the AFM stage was used to maintain the physiological temperature of 37 °C during the indentation. For every sample, 3–5 cells were indented overall, and force curves were collected. For measuring nuclear stiffness, the area of the nucleus was selected for the acquisition of force curves. One-way ANOVA with Bonferroni multiple comparison test was performed to calculate P-values at a 95 % confidence interval (n = 3; ∗∗∗P < 0.0001).

For imaging the enhanced imaging QI mode of the AFM and pyramidal tip MLCT-D silicon nitride cantilevers were used (spring constant 0.03 N/m and resonance frequency 15 kHz), and the following parameters were taken: set point 1 nN, z-length 2500 nm, pixel time 40 ms. The acquiring area of the grid was set according to the cell size, and the pixel ratio was 256 ✕ 256. The data were analyzed using JPK data processing software. The stiffness/elastic modulus of the cells (Young's modulus) was calculated with the Hertzian contact model according to Ref. [25]:

$$E=\frac{3}{4}∙\frac{F∙\left(1-\upsilon^{2}\right)}{\sqrt{\delta^{3}}∙\sqrt{R}}$$

## 3 Results

### 3.1 Design, characterization and toxicological profile of a nanofiber breast cancer *in-vitro* model

#### 3.1.1 Optimized electrospinning set-up enables mimicry of TACS5 and TACS6 architecture

During electrospinning, the polymer jet from the nozzle experiences physico-chemical instabilities [26,27], causing random fiber deposition on the collector (*e.g.* plate collector). These instabilities can be mitigated by using a rotating cylinder instead of a static collector, resulting in straight, parallel fibers.

For cell culture inserts, glass coverslips were mounted on the collectors and coated with fibers during spinning (Supplementary data, Fig. S1a). Scanning electron microscopy (SEM) images of fiber-coated coverslips (FCCs) revealed strong fiber alignment and straightening (“TACS5-like structures”) for samples spun with the rotating cylinder compared to random deposited fibers (“TACS6-like structures”) obtained from the plate collector (Fig. 1a/b). Image analysis confirmed efficient alignment (equally colored fibers) of TACS5-like structures (Fig. 1b) with a narrow peak angle distribution of 30° and an intensity of 80,000, versus the chaotic deposition of TACS6-like structures (Fig. 1a, (baseline: 7,000; maximum: 15,000). Both setups had similar fiber diameters and inter-fiber distances (Fig. 1c/d), with unaligned fibers slightly larger at 0.760 μm and 2.405 μm compared to 0.664 μm and 2.022 μm for aligned fibers, respectively. Thus, fiber orientation was adjusted without significantly affecting individual fiber characteristics.

#### 3.1.2 Collagen coating of fibers improves biocompatibility and cellular spreading on TACS-mimics

The possible toxic effects of residual solvents or incompatibilities on cell growth were evaluated. GC analysis showed complete evaporation of DCM (below 10 ng) and negligible DMF residues (1.65 ng/mg), making it unlikely for residual solvents to affect cell viability (Fig. 1e) [28]. However, PCL is highly hydrophobic and unsuitable for cell attachment. To improve tissue mimicry and enhance cellular attachment, collagen coatings were applied. Confocal microscopy confirmed effective collagen deposition on fibers (Fig. S1b).

Biological compatibility of the *in vitro* model was assessed using a fluorescence-based cell viability assay. Fig. S1c shows that cell viability on coated fibers remained high (4.6 % dead cells), with collagen coating enabling widespread cellular infiltration. Without coating, toxicity tripled to 15.1 %, with cells clustering and indicating poor matrix infiltration. Thus, the hydrophobic nature of the PCL fiber surface, not solvent residues, threatened cell viability. Due to the improved compatibility and bio-similarity of coated fibers, collagen coatings were used in subsequent evaluations.

### 3.2 *In vitro* evaluation of the bio-mimetic model

#### 3.2.1 TACS5-mimicking topographies induce EMT-like changes

In a previous *in vitro* breast cancer study, our group established an EMT phenotyping system based on EMT-marker expression, morphological changes, and cellular motility. MCF7 and HCC1954 cell lines were designated EMT-negative, MDA-MB-468 as E/M-hybrid, and MDA-MB-231 as EMT-positive. Significant changes in cellular and nuclear morphologies, such as aspect ratio (*A*_*R*_) and nuclear circularity (*C*_*N*_), predicted EMT-like changes on the protein level during growth factor-dependent EMT [22].

**Fig. 2:**
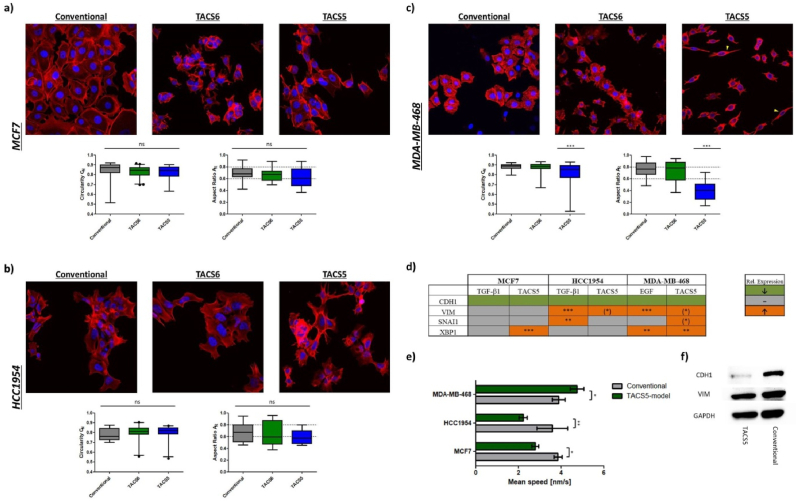
Confocal images (63×) and morphological analysis of (a) MCF7, (b) HCC1954 and (c) MDA-MB-468 cells growing on conventional cell culture dishes and TACS-like scaffolds. The blue fluorescence (DAPI) represents the nucleus and the red fluorescence (Rhodamine-Phalloidin) depicts the actin cytoskeleton. Yellow arrows indicated mesenchymal-like morphologies. The graphs below demonstrate the morphological features, nuclear circularity C_N_ (left) and aspect ratio A_R_ (right), which were analyzed and calculated with the Fiji image software and plotted as Whiskers plot in GraphPad Prism 5. (d) Schematic representation of relative mRNA-expression of EMT-relevant markers (CDH1, VIM, SNAI1, XBP1) in MCF7, HCC1954 and MDA-MB-468 cells after 72 h incubation with growth factors or growth on TACS5-mimics. Colors define changes in relative mRNA expression as shown in the right box (green: down; grey: unchanged; orange: up). (e) Mean migration speed of the latter three cell lines was recorded either on conventional coverslips or on TACS5-mimics. Means were calculated based on three individual videos with n (cells) > 50. Errors indicate standard deviation (SD). (f) Western blot of MDA-MB-468 cells stained for CDH1 and VIM after 72 h incubation on conventional coverslips or TACS5-mimics. Stars indicate statistical significance (∗∗∗P < 0.001; ∗∗P < 0.01; ∗P < 0.05). Stars in brackets (∗) indicate statistical significance after exclusion of 1 out of 4 biological replicates.

Morphologically, the three studied cell lines remained unaffected when grown on TACS6-like structures as revealed by confocal imaging (Fig. 2a–c). Similarly, growth on TACS5-mimicking scaffold did not induce prominent changes in *A*_*R*_ and *C*_*N*_*,* neither in MCF7 nor in HCC1954 cells (Fig. 2a/b). However, TACS5-like fibers caused a significant (P < 0.0001) decrease of *A*_*R*_ and *C*_*N*_ in MDA-MB-468 cells, indicating pronounced cellular alignment on the scaffold. Cancer cells dispersed within the scaffold in contrast to conventional 2D-cell culture and TACS6-like scaffolds where cells formed sheet-like aggregates. On TACS5-like scaffolds, however, cells lost their cobblestone-like morphology (*A*_*R*_ = 0.7540 ± 0.1355) and stretched into spindle-like shape (*A*_*R*_ = 0.4063 ± 0.1727) (Fig. 2c). Similarly, EMT-positive MDA-MB-231 cells aligned with fiber orientation, implying a correlation between EMT-status and cellular alignment (Fig. S2a). Significant *C*_*N*_ alterations were only observed in MDA-MB-468 and MDA-MB-231 cells.

To correlate morphological changes on TACS5-like fibers with EMT-related gene expression, qPCR analysis was performed, also after growth factor treatments known to induce EMT-like changes [22]. Expression levels of E-cadherin (*CDH1*) and Vimentin (*VIM*) and mRNA levels of the EMT transcription factors *SNAI1* and *XBP1* (X-box binding protein 1) were monitored. Snail is known to downregulate CDH1 and upregulate VIM [29], while XBP1 has been recently linked to EMT and metastasis, impacting cancer progression and therapy outcomes [30].

Fig. 2d summarizes relative mRNA expression (absolute values in Fig. S2b) with green indicating downregulation and orange representing upregulation of gene expression. TACS5-like topographies decisively influenced gene expression depending on the cell line, with accessibility to contact guidance cues increasing with EMT-status (MCF7 < HCC1954 < MDA-MB-468).

Overall, CDH1 expression was equally downregulated across cell line and stimulatory cue. Fiber topography induced significant upregulation of *XBP1* (P < 0.001) in MCF7, VIM (≈1.7 fold) in HCC1954, and *XBP1* (≈2.3 fold, P < 0.01), *SNAI1* (≈2.1 fold) and *VIM* (≈1.9 fold) expression in MDA-MB-468. Western blotting confirmed topography-induced downregulation of CDH1 protein levels in MDA-MB-468, while VIM protein levels remained unaffected (Fig. 2f). Although similar EMT-related genes were affected by contact guidance, growth factor-mediated mRNA expression changes were 2–3 magnitudes higher (Fig. S2b).

Another feature comprised in EMT-like changes is accelerated cellular motility. Live cell imaging estimated mean migration speed on conventional coverslips and TACS5-mimicking scaffolds. Fig. 2e demonstrates that TACS5-like topographies strongly influenced random cell migration. Mean speeds of MCF7 and HCC1954 cells decreased by 28 % and 38 %, respectively, while velocity of MDA-MB-468 significantly increased by 22 % on the fibrous matrix. Differences in motility and contact guidance among all four cell lines on the TACS5 model were measured. Besides mean migration speed, three trajectory features indicative of directional persistence were analyzed (see Table 1). Directional persistence increases with higher track displacement and confinement ratio but decreases with mean directional change. MDA-MB-468 and MDA-MB-231 displaced significantly further (46 % and 144 %) than MCF7 and HCC1954 over 24 h (Fig. S2c). Fig. S2e shows that MDA-MB-468 cells moved fastest (4.76 nm/s), followed by MDA-MB-231 (3.32 nm/s), MCF7 (2.67 nm/s) and HCC1954 (2.12 nm/s). MDA-MB-231 cells migrated most efficiently along the fibers, with the highest confinement ratio and the lowest mean directional change (Fig. S2d/f).

#### 3.2.2 TGF-β stimulation enhances contact guidance on TACS5-mimicking scaffolds and topographical cues potentiate EMT in HCC1954

**Fig. 3:**
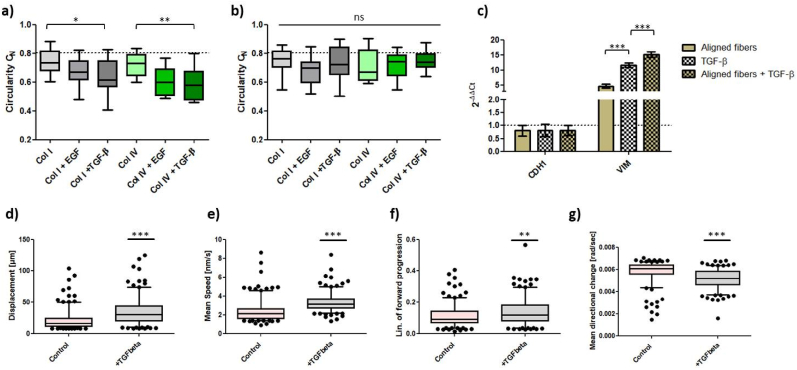
Morphological alignment of nuclei of HCC1954 cells on (a) TACS5- and (b) TACS6-like scaffolds depending on growth factor stimulation (EGF, TGF-β1) and growth surfaces (collagen I/IV). C_N_-values were plotted as Whiskers plot. (a, b) The dotted line represents the mean C_N_-value of control cells growing on conventional culture dishes. (c) Relative mRNA expression of CDH1 and VIM in HCC1954 cells. ΔΔC_t_-values were calculated after 72 h of either growth on TACS5-mimics and/or treatment with 10 ng/mL TGF- β1 (n = 2). Error bars depict SD. (d-g) Comparison of trajectory analysis of untreated (control) HCC1954 cells and cells treated with TGF-β1 using Fiji software (TrackMate). Displacement, mean migration speed, confinement ration and mean directional change were plotted as whiskers plot (5–95 percentiles). Statistical significance was assessed with a *t*-test based on > 200 cells/condition. Stars indicate statistical significance (∗∗∗P < 0.001; ∗∗P < 0.01; ∗P < 0.05, ns = not significant).

Cancer cells gain phenotypic plasticity during EMT-like changes, enhancing cell-substrate interactions within the TME [31]. Efficient migration through a porous matrix requires nuclear deformation, as the nucleus is the greatest cell organelle and dictates cellular migration and invasion in cancer [32,33]. Since MCF7 and HCC1954 cell migration slowed down on TACS5-mimicking scaffolds, without elongation of cellular and nuclear morphologies (Fig. 2a/b), EMT was induced using growth factors (EGF, TGF-β1) and ECM components (collagen I/IV), promoting cancer cell spreading and alignment within the scaffolds (Fig. S3a/b). Growth factor treatment reduced cell-cell contacts (green arrows) and increased membrane protrusion (yellow arrows) prominently, fostering a mesenchymal-like morphology (yellow arrows) on TACS5-like mimics, especially in TGF-β1-treated HCC1954 cells. This led to alignment with fiber orientation on the TACS5-mimicking scaffolds and significantly decreased *C*_*N*_ values on TACS5-like (Fig. 3a) but not TACS6-like structures (Fig. 3b) indicating the importance of unidirectional fiber orientation and growth factor treatment. Similar results were observed in the MCF7 cell line (Fig. S3a), where growth factors enhanced cell-fiber interaction and promoted mesenchymal-like morphologies (yellow arrows) in comparison to untreated cells clustered in sheet-like aggregates (green circle).

To evaluate combinatorial cue effects, mRNA expression of CDH1 and VIM in HCC1954 was analyzed. Fig. 3c displays equally downregulated CDH1 mRNA levels (18–20 %) among the three conditions. However, the combined treatment significantly increased VIM expression, showing a 15.2-fold increase compared to a 11.6-fold increase upon TGF-β1 treatment alone. These results demonstrated that TACS5-mimicking scaffolds alone can induce EMT-like changes in HCC1954 cells which is fostered by combinatorial action with GFs.

Trajectory analysis (Fig. 3d–g) revealed that TGF-β1-induced EMT significantly affected HCC1954 migration on TACS5-scaffolds. Cells migrated 70.5 % further and 39.3 % faster than control cells. With a 23 % increase in confinement ration and 12 % decrease in mean directional change, cells migrated more efficiently on the fibrous matrix, indicating reinforced contact guidance.

#### 3.2.3 Mesenchymal-to-epithelial-transition (MET)-like changes impair motility and contact guidance on TACS5-mimicking scaffolds

The data indicated a reciprocal dependence between topographical cue and cancer phenotype. Growth factor-induced EMT-like changes enhanced cancer cells’ ability to use topographical input for cellular migration. To further confirm this observation and explore if MET-like changes reverse these effects, two genetically modified breast cancer cell lines were studied: miR200c knock-out MCF7 cells (EMT cell line), and miR200c-inducible expression in MDA-MB-231 cells (MET cell line) under doxycycline (DOX) exposure [34].

**Fig. 4:**
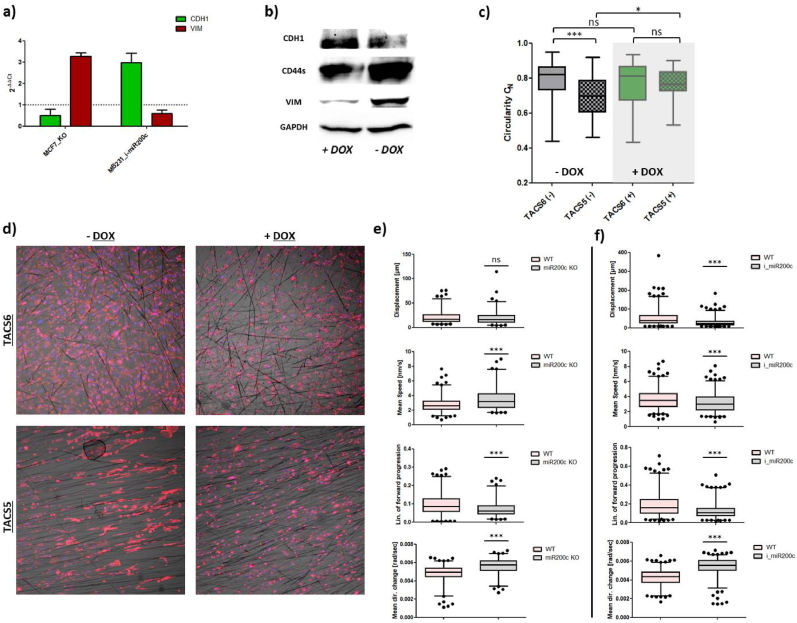
(a) Relative expression of CDH1 and VIM mRNA in MCF7_miR200c_KO and DOX-treated MDA-MB-231_i-miR200c (MB231_i-miR200c) cells normalized to wild type (unmodified MCF7 or untreated MB231_i-miR220c, respectively). (b) Western blot of MB231_i-miR200c cells with and without doxycycline-induction stained for EMT-relevant marker CDH1, VIM and CD44s. (c) Whiskers plot showing alterations in C_N_-values (from 40× images) in MB231_i-miR200c cells with and without doxycycline-induction for growth on TACS5- and TACS6-like structures. One-way ANOVA with Bonferroni multiple comparison test was performed to calculate P-values at a 95 % confidence interval (n (cells) > 50). (d) Corresponding confocal images of (c) at 10× magnification. Cells were stained for nuclei (blue) and the cytoskeleton (red). Comparison of trajectory analysis of (e) WT cells with MCF7_KO or (f) MB231_i-miR200c, respectively, performed with Fiji software (TrackMate). Displacement, mean migration speed, confinement ration and mean directional change were plotted as whiskers plot (5–95 percentiles). Statistical significance was assessed with a *t*-test based on > 150 cells/condition. Stars indicate statistical significance (∗∗∗P < 0.001; ∗∗P < 0.01; ∗P < 0.05, ns = not significant).

First, CDH1 and VIM mRNA levels were measured in unmodified MCF7 cells (MCF7_WT), miR200c knock-out MCF7 (MCF7_KO), and inducible MDA-MB-231 (MB231_i-miR200c) cells with and without DOX stimulation. MiR200c knock-out in MCF7 decreased CDH1 expression by 50 % and increased VIM mRNA levels 3.2-fold. DOX-induced miR200c expression in MDA-MB-231 cells increased CDH1 mRNA 3.0-fold and decreased VIM expression by 40 % (Fig. 4a). CDH1 and VIM protein levels changed similarly, and CD44s protein levels, overexpressed in cancer stem cells and during EMT [17,35], were partially depleted upon DOX stimulation indicating MET (Fig. 4b).

Second, nuclear circularity and cell growth of MB231_i-miR200c cells in TACS5- and TACS6-mimicking scaffolds were monitored. After 48 h, untreated cells aligned with fiber orientation (compare Fig. S2a), while DOX-induced miR200c expression impeded morphological polarization on aligned fibers (Fig. 4d). *C*_*N*_ analysis confirmed this with elevated values for DOX-treated cells and no significant differences between TACS5 and TACS6-like scaffolds (Fig. 4c).

Third, live cell imaging-based trajectory analysis evaluated the effect of EMT-like (MCF7_KO) or MET-like (MB231_i-miR200c) changes on cellular motility on TACS5 mimics. miR200c KO increased migration speed significantly (P < 0.0001) but did not improve contact guidance; track displacement remained unchanged, confinement ratio decreased (P < 0.001), and mean directional change increased (P < 0.001) (Fig. 4e). Conversely, miR200c induction in MDA-MB-231 cells reduced mean speed (WT: 3.625 ± 0.068 nm/s vs. i_miR200c: 3.151 ± 0.064 nm/s) and impaired contact guidance, affecting track displacement, confinement ratio and mean directional change (Fig. 4f). Overall, MET-like changes restricted contact guidance by TACS-like structures.

### 3.3 Biomechanical evaluation of breast cancer cell lines: comparison of conventional 2D cell culture with TACS5-mimicking *in vitro* model

#### 3.3.1 EMT-status inversely correlates with total cellular stiffness

**Fig. 5:**
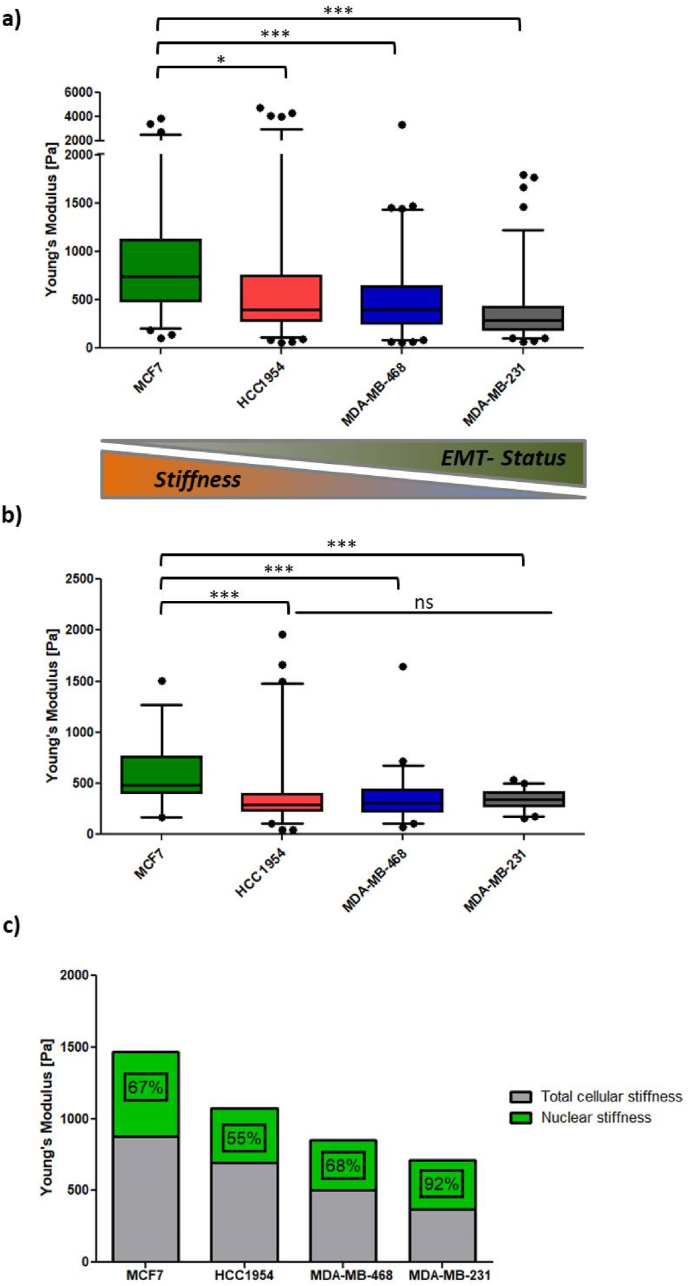
(a) Total cellular stiffness of the 4 breast cancer cell lines shown as Whiskers plot (5–95 percentiles). (b) Nuclear stiffness of the 4 breast cancer cell lines shown as Whiskers plot (5–95 percentiles). (c) Nuclear stiffness stacked over total cellular stiffness. Numbers indicate contribution (in percent) of nuclear stiffness on total cellular stiffness. Impact of nuclear stiffness on total nuclear stiffness increases with EMT-status. Stars indicate statistical significance (∗∗∗P < 0.001; ∗P < 0.05; ns = not significant).

Total cellular stiffness and nuclear stiffness of single cells were estimated using atomic force microscopy (AFM) on either the whole cell or the nucleus. The Elastic Young's modulus [Pa], a good approximation of cellular stiffness [36,37], was quantified. Cell mechanics varied significantly among the four cell lines. As demonstrated in Fig. 5a, total cellular stiffness decreased with EMT-status, from 769.5 ± 54.5 Pa (MCF7) to 372.1 ± 20.8 Pa (MDA-MB-231). This trend was not observed for nuclear stiffness. While MCF7 cells had the highest Young's modulus (590.0 ± 280.0 Pa), the nuclear stiffness of the other three cell lines did not differ significantly (Fig. 5b). However, in Fig. 5c, nuclear stiffness of the most invasive MDA-MB-231 cells contributed to the overall stiffness (92 %), whereas this contribution was less important for the other three cell lines (55–68 %). This indicates that the nucleus of this mesenchymal cell line is the main physical restriction for invasion. Lamins (A/C, B), nuclear envelope proteins, are thought to determine nuclear physical properties. Contrary to the literature [38,39], high levels of Lamin B1, instead of Lamin A/C, correlated with increased nuclear stiffness in the studied breast cancer cell lines (Supplementary data Figure S4).

#### 3.3.2 Early EMT-like events entail cellular/cytoskeletal stiffening during biochemically and topographically induced EMT

**Fig. 6:**
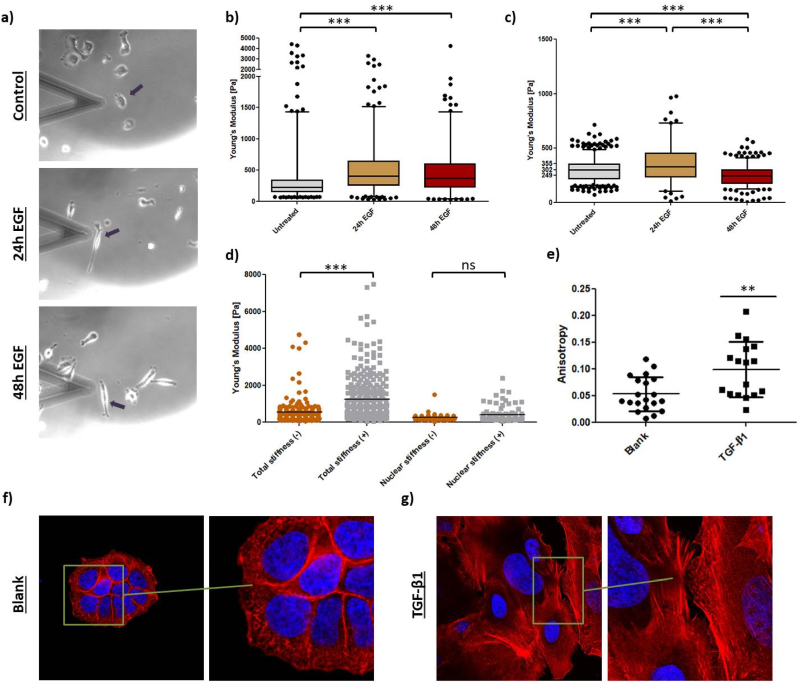
(a) Transmitted light photography images of MDA-MB-468 cells measured in AFM contact mode. Black arrows indicate analyzed cell. (b) Total cellular stiffness of MDA-MB-468 cells treated with EGF for 24 and 48 h. (c) Nuclear stiffness of MDA-MB-468 cells treated with EGF for 24 and 48 h (c). Total cellular stiffness of HCC1954 cells treated with TGF-β1 for 48 h. (d) Nuclear stiffness of MDA-MB-468 cells treated with TGF-β1 for 48 h. (e) Anisotropy of actin fibers of control HCC1954 cells compared with TGF-β1 stimulated cells (*t*-test; ∗∗P < 0.01). Confocal images of (f) untreated and (g) TGF-β1-treated HCC1954 cells showing nuclei (blue) and actin cytoskeleton (red) at 63× magnification. Right panel (zoom-in) highlights actin cytoskeleton architecture to either show a cross-linked network (f) or parallel stress fibers (g). Stars indicate statistical significance (∗∗∗P < 0.001; ns = not significant).

These findings imply that EMT induction would decrease total cellular stiffness and nuclear stiffness. To challenge this hypothesis, MDA-MB-468 cells were stimulated with EGF for 24 h or 48 h to undergo EMT, and their biomechanical properties were analyzed (Fig. 6a–c). After 24 h, epithelial cancer cells adopted a spindle-like morphology (Fig. 6a). Contrary to expectations, total cellular stiffness significantly increased upon EGF-treatment, with Young's moduli rising by 39.6 % and 28.9 %, respectively (Fig. 6b). Nuclear stiffness also increased by 17.6 % within the first 24 h. However, after 48 h, the Young's modulus decreased by 21.4 % below the values of control cells, indicating nuclear softening (Fig. 6c).

To validate these findings, EMT was also induced in HCC1954 cells. Confocal scanning microscopy was used to monitor changes in the actin cytoskeleton, which is key to cell elasticity. Total cellular stiffness significantly increased upon TGF-β1 stimulation (Fig. 6d), with the Young's modulus rising by 117.9 % (from 564.4 Pa to 1230.0 Pa), surpassing the EGF-induced increase in MDA-MB-468 cells. However, nuclear stiffness did not change significantly upon TGF-β1 treatment. Image analysis illustrated cytoskeletal rearrangements explaining the dramatic cell stiffening. Control cells had a crosslinked, randomly orientated actin network (Fig. 6f), while TGF-β1-induced EMT elongated HCC1954 cells and restructured actin bundles into stress fibers (Fig. 6g). Actin fibers became significantly more parallel compared to the network-like structure in control cells, indicated by increased anisotropy (Fig. 6e).

**Fig. 7:**
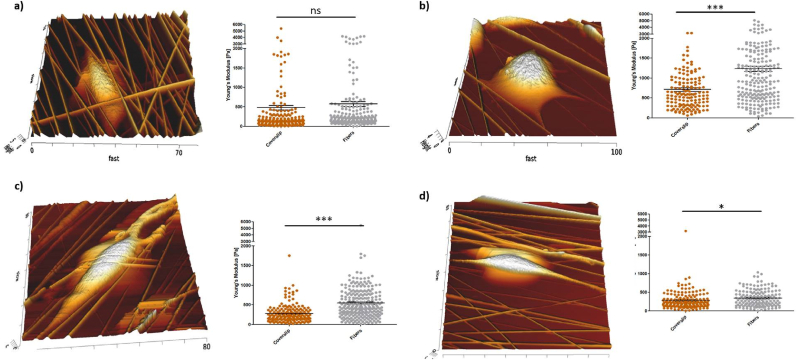
AFM analysis of (a) MCF7, (b) HCC1954, (c) MDA-MB-468 and (d) MDA-MB-231 cell growth on TACS5-like scaffolds. Left panel shows surface topography. Right panel depicts total cellular stiffness of the respective cell lines growing either on conventional culture dishes or TACS5-mimics. Stars indicate statistical significance (*t*-test: ∗∗∗P < 0.001; ∗P < 0.05; ns = not significant).

Next, the effect of cell growth on TACS5-topographies on biomechanical changes was assessed to correlate EMT-marker expression changes with cell mechanics. Cellular stiffness was compared between cells growing on collagen I-coated coverslip and coated fibers. The Young's modulus remained unaffected in MCF7 cells (Fig. 7a) and slightly increased in MDA-MB-231 cells (Fig. 7d) on TACS5-mimicking scaffold. Interestingly, a significant increase (P < 0.0001) in cellular stiffness occurred in HCC1954 (Fig. 7b) and MDA-MB-468 (Fig. 7c) cells growing on the fibrous matrix, with Young's moduli increasing by 72.9 % and 95.2 %, respectively, similar to GF-mediated EMT induction.

## 4 Discussion

The goal of this *in vitro* study was to create a reproducible 3D electrospun matrix that mimics the topography and dimension of tumor-associated collagen signatures found in diseased mammary gland tissue. Two electrospinning collector types, namely a rotating mandrel and a plate collector, were used to mimic TACS5 or TACS6 structures, respectively. SEM images revealed that optimized PCL fibers on coverslips had either a parallel (mandrel) or a random (plate) orientation. Fiber diameters ranged from 400 to 1700 nm with comparable mean inter-fiber distances. Naturally occurring collagen structures have widths from a few hundred nanometers to one micron [40] and inter-fiber distances in breast cancer tissues range from 2 μm to 3 μm [41]. The optimized electrospinning setup imitated the *in vivo* architecture of collagen fibers by producing scaffolds with TACS5 and TACS6 orientations and appropriate submicron scale and fiber density. With negligible cytotoxicity on collagen-coated fibers, the *in vitro* model was successfully established.

Several earlier studies demonstrated that breast cancer cells acquired a more malignant phenotype with EMT-like changes on fibrous 3D scaffolds compared to conventional 2D cell culture [[42], [43], [44], [45], [46]]. This response to topographical cues, known as contact guidance, includes cellular polarization, alignment, altered cell motility, and gene expression. The transition magnitude depends on the fiber matrix’ physical characteristics and biological aspects of the cells, including scale (nanometric vs. micrometric), topography orientation (unidirectional vs. multidirectional), cell size and type, and the relation between cell size and substrate dimension [[42], [43], [44], [45], [46], [47], [48]]. Additionally, the epithelial/mesenchymal-state of a cell influences the degree of contact guidance [15]. This study found that cells with low EMT-status had weak contact guidance on the TACS5-mimicking model, while high EMT status related to strong guidance. TACS6-like structures did not provoke significant morphological changes. Cells were also described to align and migrate directionally on TACS5-like substrates but orientate randomly and migrate on multidirectionally on TACS6-like patterns [48].

In case of MDA-MB-468 cells on TACS5-mimics (Fig. 2, Fig. S2), cytoskeletal and nuclear morphology changes coincided with EMT-phenotypic gene expression pattern (↓CDH1, ↑VIM, ↑SNAI, ↑XBP1, Fig. 2d), highlighting the predictive importance of (sub-)cellular morphologies. In contrast, MCF7 cells, which did not significantly change morphology, retained their epithelial expression pattern (Fig. 2a/d). Migration on TACS5-like scaffolds was slower in case of MCF7 and HCC1954 cells, while mesenchymal MDA-MB-468 cells demonstrated increased migration speed (Fig. 2e). The EMT-status was crucial in determining cellular responses, with cells having a partial mesenchymal phenotype showing polarization, pro-EMT transcription, and increased motility.

TGF-β1-mediated downregulation of CDH1 and upregulation of VIM in HCC1954 supported alignment and migration within the TACS5-like fiber matrix (Fig. 3a, Fig. S3b). This increased scaffold interaction fostered mesenchymal transcription (Fig. 3c). As shown in Fig. 2 and Fig. S3 epithelial phenotypes in the TACS5-micking matrix tend to establish cell-cell contacts, while mesenchymal phenotypes interact more with the matrix. Focal adhesion-mediated recognition of topographical patterns likely induced cell polarization and actomyosin contractility, modulating EMT-related gene transcription by affecting chromatin arrangement [[49], [50], [51]]. Ravikrishnan et al. [44] demonstrated that HGF-dependent EMT-induction was necessary for epithelial cells to disintegrate, interact with, and migrate along nanofibers. E-Cadherin-based adherens junctions may have restrained contact guidance by counteracting anisotropic cell-substrate interaction [15]. Additionally, the absence of vimentin in fully epithelial cells may explain impaired contact guidance, as vimentin aligns with grooved patterns in meningeal cells [52] and affects focal adhesion maturation [53,54]. Since vimentin templates microtubule-mediated directional migration [55], its presence and upregulation enhances contact guidance. These findings align with observations that miR200c-dependent MET induction in MDA-MB-231 cells downregulates vimentin, significantly reducing contact guidance on TACS5-like topographies (Fig. 7). MET induction restricted cellular alignment and directed migration. Conversely, miR200c KO in MCF7 (EMT-induction) only marginally elevated contact guidance, likely because CDH1 mRNA expression still dominated over VIM expression.

Biochemically induced EMT resulted in stronger overall EMT-like changes than topographical triggers within the 72 h (Fig. 5a, Fig. S2b), corroborating findings by Saha et al. [43] regarding EMT-relevant genes affected by contact guidance. Moreover, to the best of our knowledge, the present study is the first report to link XBP1 to contact guidance, with significant upregulation in two of three cell lines after 72 h of cell growth on the fibers (Fig. S2b). XBP1, known to upregulate SNAI1 expression and induce EMT-like changes [30,56], was upregulated alongside SNAI1 in the MDA-MB-468 cell line. Future studies should clarify XPB1's role in contact guidance, potentially involving ER stress and unfolded protein response (UPR) due to spatial confinements, which could provide survival advantage in cancer cells [57].

Cell mechanical properties are defined by the nucleus, the actin cytoskeleton, microtubules, intermediate filaments and cell membrane, and are altered by interactions with adjacent cells and the ECM [58]. EMT-like changes reorganize the cytoskeleton, affecting cell mechanics.

AFM indentation revealed that Young's moduli of total cellular stiffness decreased with increasing EMT-status (MCF7 > HCC1954 > MDA-MB-468 > MDA-MB-231, Fig. 5a), aligning with cancer cell malignancy [[58], [59], [60], [61], [62], [63]]. However, probing nuclear stiffness revealed some inconsistencies. In line with earlier studies nuclei were less compliant in highly invasive MDA-MB-468 and MDA-MB-231 cells compared to poorly invasive MCF7 cells [32]. Contrary to literature [33], nuclei were not stiffer than the surrounding cytoskeleton.

Changes in cell mechanics during EMT were also investigated. While some studies report cell stiffening during GF-mediated EMT [64,65], others observed softening [[66], [67], [68]]. This study supports cell stiffening within 24–48 h of GF induction in two cell lines (Fig. 6a–d). Phalloidin staining of HCC1954 cells demonstrated strongly aligned actin bundles under TGF-β1 stimulation (Fig. 6e–g), correlating stress fibers with increased stiffness [[69], [70], [71], [72]]. Moreover, Tavares et al. [72] revealed pre-metastatic cell stiffening before converting into a malignant but softer phenotype. Nuclear stiffness changes in MDA-MB-468 cells also supported initial stiffening followed by softening, highlighting the importance of transition time in biomechanical EMT evaluations.

Vimentin, proposed to maintain mechanical integrity during invasion, correlated with cell stiffening in lung and breast cancer cells [64,73,74]. However, vimentin expression did not directly correlate with cell stiffness in the studied breast cancer cell lines (Fig. 5a). MDA-MB-231 cells, with the highest vimentin levels [22], exhibited the lowest Young's modulus. Further studies on vimentin network assembly and cellular stiffness are needed to better understand context-dependent cell mechanics Presumably, as proposed elsewhere, geometry and organization of the vimentin network, which changes upon EMT, as well as interactions with other cells dictate vimentin's contribution to overall cell mechanics, which seems to be ultimately context-dependent [73,75].

Cellular mechanics on TACS5 scaffolds conformed to prior findings. MCF7 cells showed no stiffness change in comparison to 2D cell culture (Fig. 7a), while already polarized MDA-MB-231 cells [22] slightly stiffened. HCC1954 and MDA-MB-468 cells, undergoing EMT-like changes on the TACS5-like scaffold, significantly increased stiffness (Fig. 7b and c), similar to biochemically induced EMT. This suggests transient stiffening of breast cancer cells as a new marker for EMT-like changes. In future experiments, longer time intervals of the biomechanical evaluation need to be assessed to further study the kinetics of cellular mechanics of the EMT process. Ideally, understanding and interrupting the mechanical adaption may prevent tumor cell dissemination [64].

Discrepancies with existing literature may stem from different biophysical techniques, culture dishes or probing parameters [37]. Examining single-cell versus epithelial layer mechanics [67,74] yields distinct outcomes due to cytoskeleton reorganization. AFM parameters likely caused observed deviations, as the cytoskeleton contribution to nuclear stiffness was negligible.

## 5 Conclusion

This *in vitro* study provided strong evidence that TACS5-like structures support and promote EMT-like changes in breast cancer cells. Contact guidance highly depended on the EMT-status, not necessarily requiring a full transition to a mesenchymal phenotype. Epithelial depletions or mesenchymal acquisitions creating E/M-hybrids improved single-cell contact guidance of single cells. *In vivo,* EMT-phenotypic changes and restructuring of the stromal compartment's acellular fraction coincide in breast cancer. This co-action likely creates a positive feedback loop that is sustains tumor progression, ultimately leading to metastasis. A plausible strategy to inhibit this loop is to target initial transition into a (partial) mesenchymal phenotype or disturb biomechanical adaption of cancer cells. While the diagnostic value of TACS is yet to be confirmed, early detection may allow therapeutic prevention of disease progression.

## Declaration of generative AI and AI-assisted technologies in the writing process

During the preparation of this work the authors used ChatGPT in order to shorten parts of the original manuscript. After using this tool, the authors reviewed and edited the content as needed and take full responsibility for the content of the publication.

## CRediT authorship contribution statement

**Lorenz Isert:** Writing – original draft, Visualization, Software, Methodology, Investigation, Formal analysis, Data curation, Conceptualization. **Mehak Passi:** Methodology, Investigation, Formal analysis. **Benedikt Freystetter:** Methodology, Investigation. **Maximilian Grab:** Resources, Methodology, Investigation. **Andreas Roidl:** Writing – review & editing, Supervision, Resources, Methodology, Investigation, Conceptualization. **Christoph Müller:** Writing – review & editing, Resources, Methodology, Investigation, Formal analysis. **Aditi Mehta:** Writing – review & editing, Supervision, Methodology, Investigation, Conceptualization. **Harini G. Sundararaghavan:** Writing – review & editing, Supervision, Resources, Methodology, Investigation, Conceptualization. **Stefan Zahler:** Writing – review & editing, Supervision, Resources, Methodology, Conceptualization. **Olivia M. Merkel:** Writing – review & editing, Supervision, Resources, Project administration, Methodology, Funding acquisition, Conceptualization.

## Declaration of competing interest

O.M.M. acknowledges the Munich Multiscale Biofabrication Network, funded by the German 10.13039/501100002347Federal Ministry of Education and Research. A.R and S.Z were financially supported by the 10.13039/501100001659Deutsche Forschungsgemeinschaft (DFG, German Research Foundation) - Project-ID 201269156 - SFB 1032 (Projects B04 and B08). M.P was supported by the DAAD (German Academic Exchange Service). O.M.M. is a co-founder of RNhale GmbH, an SAB member for AMW GmbH, Corden Pharma International GmbH, and Coriolis Pharma GmbH and a consultant for AbbVie Deutschland GmbH and PARI Pharma GmbH on unrelated projects.

## Data Availability

Data will be made available on request.
