# Supplementary material for: Cellular EMT-status governs contact guidance in an electrospun TACS-mimicking in vitro model
Source: Mater Today Bio. 2024 Dec 10;30:101401. doi: 10.1016/j.mtbio.2024.101401 (PMC11699613; doi:10.1016/j.mtbio.2024.101401)
Supplement: Multimedia component 1 [file mmc1.docx]

**Cellular EMT-status governs contact guidance in an electrospun TACS-mimicking *in vitro* model**

# Lorenz Isert^1^, Mehak Passi^2^, Benedikt Freystetter^3^, Maximilian Grab^3^, Andreas Roidl^4^, Christoph Müller^5^, Aditi Mehta^1^, Harini G. Sundararaghavan^6^, Stefan Zahler^2^, Olivia M. Merkel^1^

^1^Pharmaceutical Technology and Biopharmaceutics, Department of Pharmacy, Ludwig-Maximilians-University München, Munich, Germany

^2^Pharmaceutical Biology, Department of Pharmacy, Ludwig-Maximilians-Universität München, Munich, Germany

^3^Department of Cardiac Surgery, Ludwig Maximilians University München, Munich, Germany

^4^Pharmaceutical Biotechnology, Department of Pharmacy, Ludwig-Maximilians-Universität München, Munich, Germany

^5^Center of Drug Research, Department of Pharmacy, Ludwig-Maximilians-Universität München, Munich, Germany

^6^Department of Biomedical Engineering, Wayne State University, Detroit, Michigan, USA

**Supplementary information**

| **Table S1:** Conditions of static HS-GC-MS | | | |
| --- | --- | --- | --- |
| **Headspace sampler parameters** | | **GC-MS parameters** | |
| Agitator cycle | 5 sec on, 2 sec off | Carrier flow rate | 1.2 mL min^-1^ |
| Agitator speed | 500 rpm | Split ratio | 10 : 1 |
| Agitator temperature | 200 °C | Oven profile | 40 °C for 6 min  50 to 240 °C at 50 °C min^-1^  (hold time 2.5 min) |
| Sample incubation time | 15 min | Transfer line temperature | 250 °C |
| Syringe size | 2.5 mL | Inlet temperature | 200 °C |
| Syringe temperature | 150 °C | Ion source temperature | 230 °C |
| Injection volume | 1.00 mL | Quadrupole temperature | 150 °C |

**
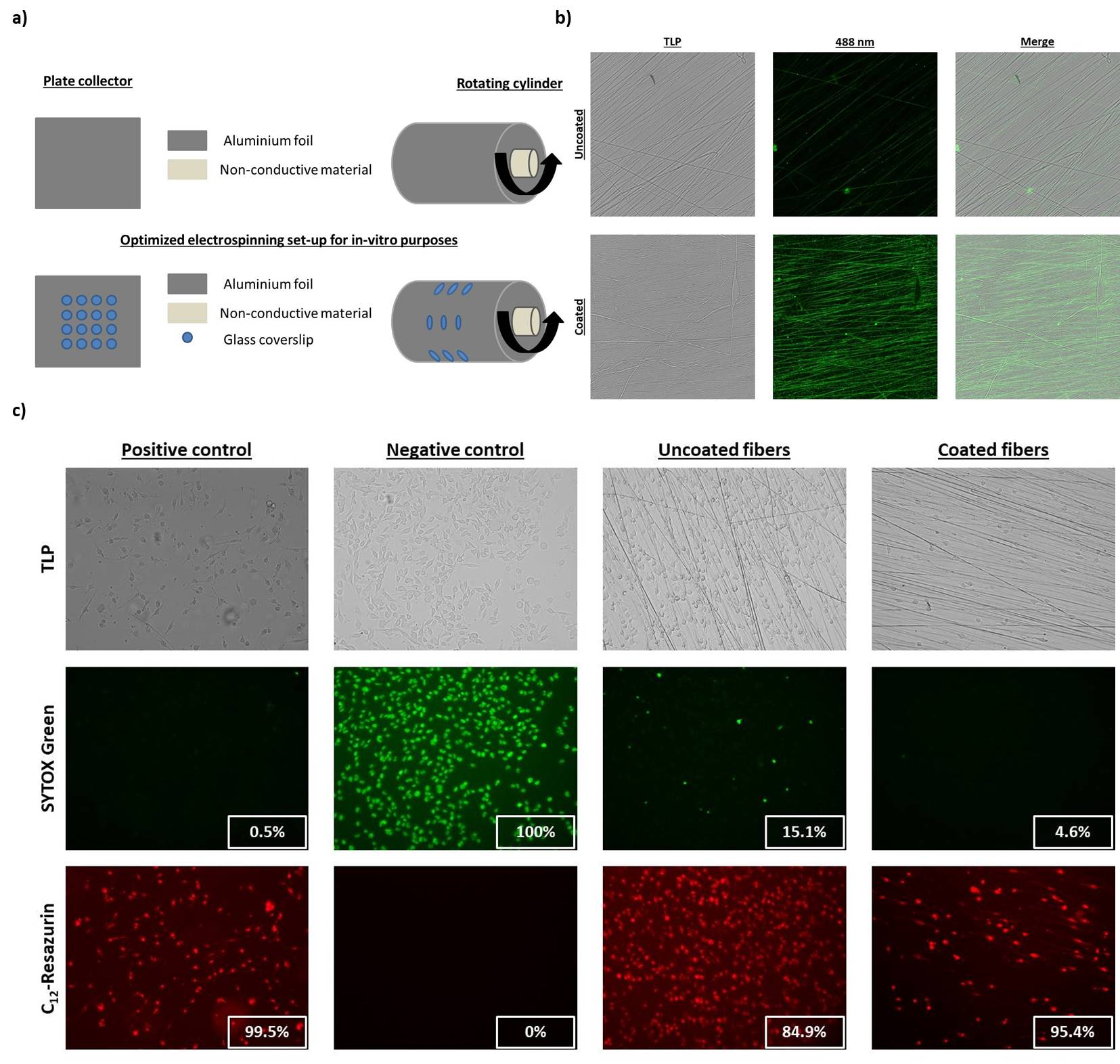

Supplementary Figure S1:** (**a**) Schematic illustration of collector types and optimized electrospinning set-up. (**b**) Evaluation of collagen coating of TACS5-sccaffolds assessed *via* confocal imaging. Upper panel shows uncoated samples and lower panel coated ones. (**c**) Comparison of cell viability on conventional culture dishes (positive control) and TACS-mimicking scaffold with and without collagen coating. Ethanol treated cell represent negative control. Upper panel: Transmitted light photography (TLP); Mid panel: Fluorescence at 488 nm (dead stain); Lower panel: Fluorescence at 595 nm (live stain). White numbers indicate number of living/dead cells [%] of total amount of cells.

**
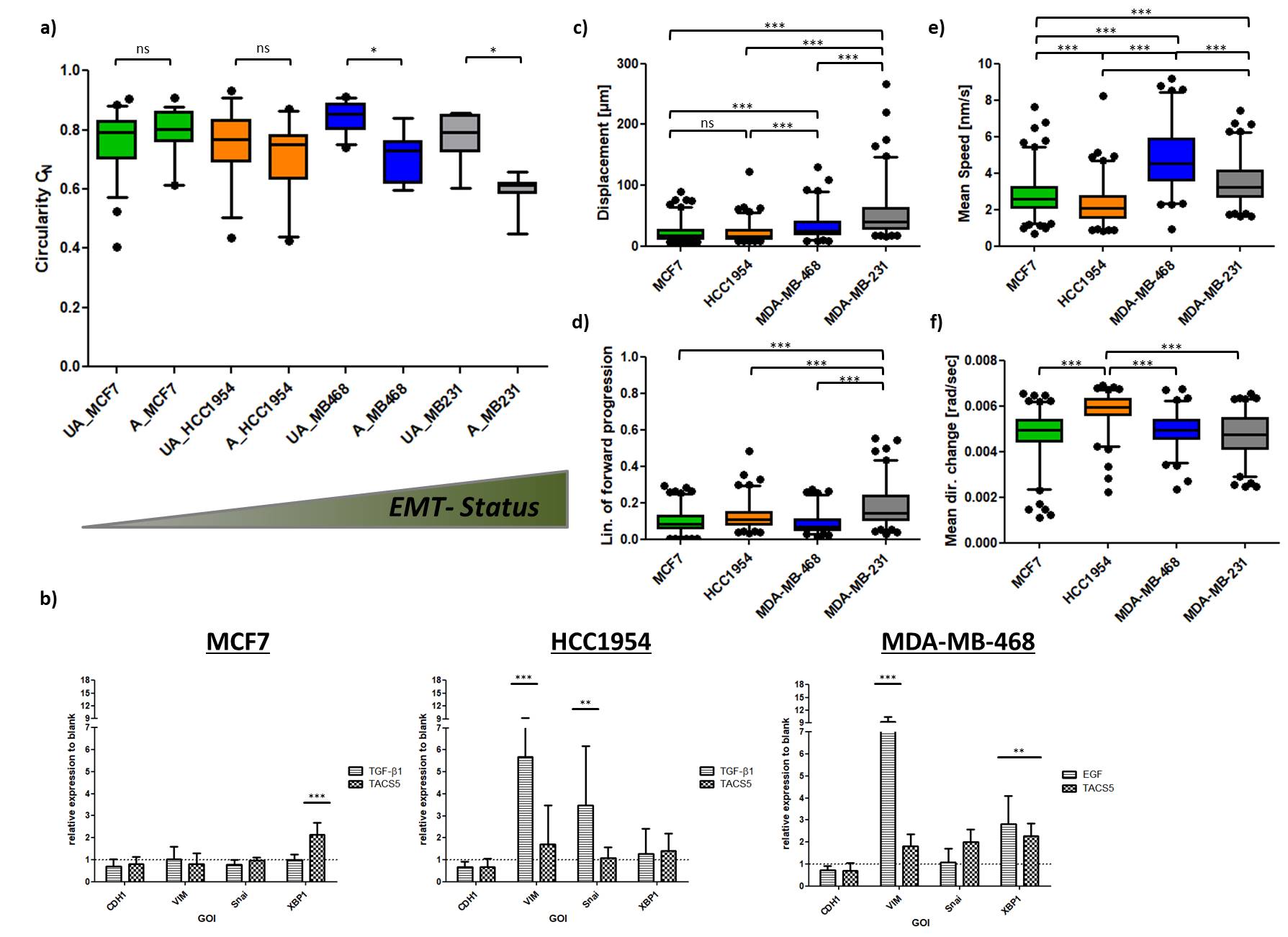

Supplementary Figure S2:** (**a**) Nuclear circularity of cancer cell lines grown on TACS5-(A) or TACS6-(UA) mimics. (**b**) Relative mRNA-expression of EMT-relevant marker (CDH1, VIM, SNAI1, XBP1) derived from MCF7, HCC1954 and MDA-MB-468 cells after 72 h incubation with growth factors or growth on TACS5-mimics. (**c-f**) Trajectory analysis of 4 breast cancer cell lines within TACS5-like fibers performed with Fiji software (TrackMate): (**c**) track displacement, (**d**) confinement ratio, (**e**) mean migration speed and (**f**) mean directional change were plotted as whiskers plot (5-95 percentiles). Stars indicate statistical significance (***P < 0.001; **P < 0.01; *P < 0.05, ns = not significant).

**
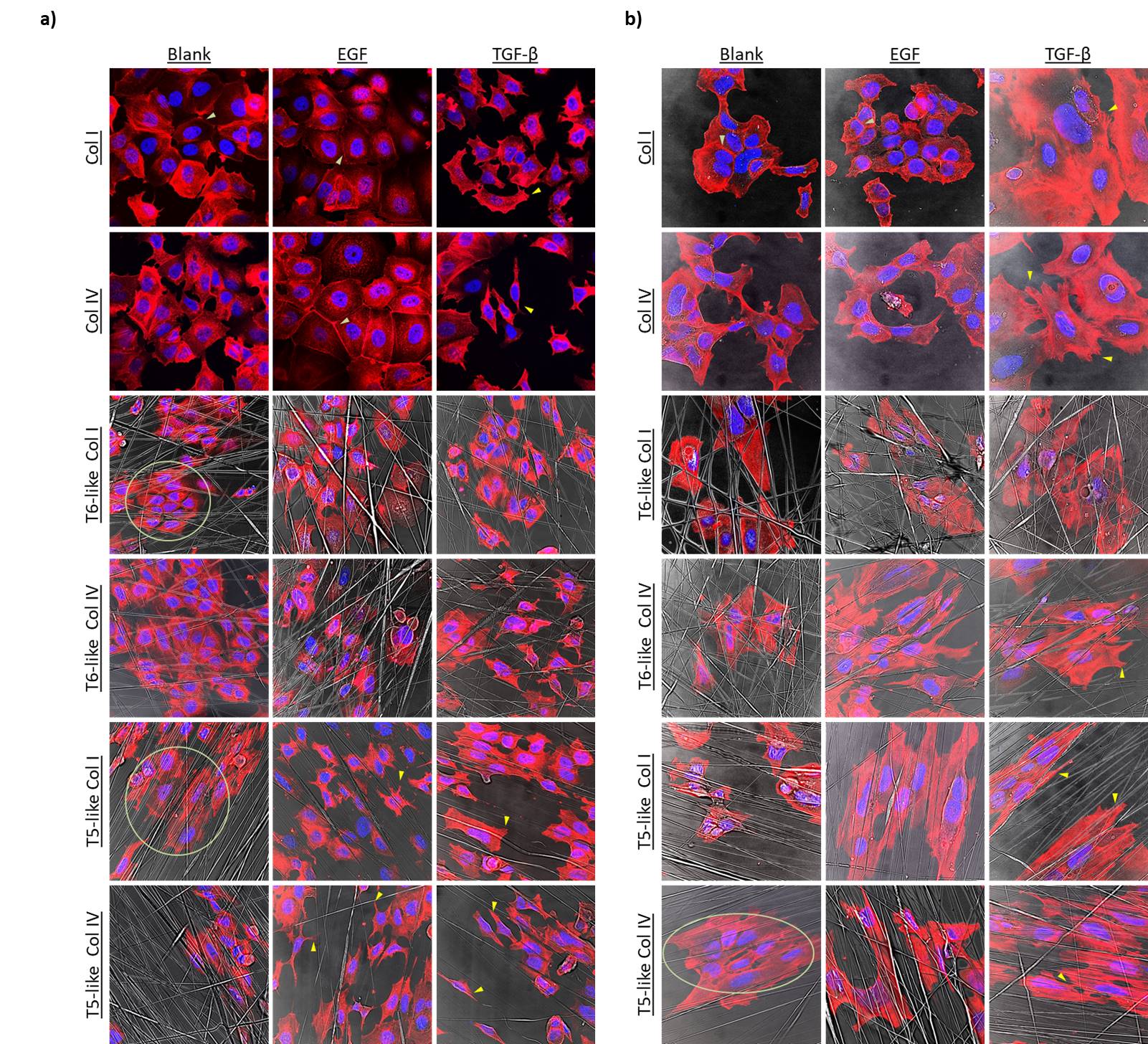
Supplementary Figure S3:** Confocal images of (**d**) MCF7 and (**e**) HCC1954 cells under various treatments/conditions. Cells were stained for nuclei (blue) and cytoskeleton (red). To show the fibrous environment of the different TACS-mimics TLP channels were included. Grey arrows/circles indicate strong cell-cell contacts and/or epithelial clustering of cancer cells. Yellow arrows emphasize mesenchymal-like morphologies/protrusions, cell-fiber interactions and/or strong cellular alignment with the scaffold.

**
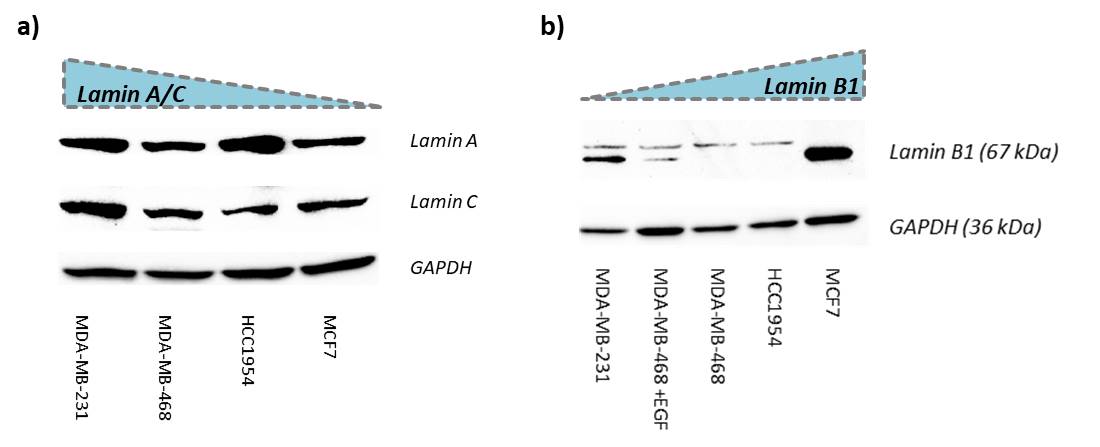

Supplementary Figure 4:** Western blots of nuclear envelop proteins (**a**) Lamin A/C and (**b**) Lamin B1.
